# Association of HLA class I and II genes with Middle East respiratory syndrome coronavirus infection in Koreans

**DOI:** 10.1002/iid3.541

**Published:** 2021-10-12

**Authors:** In‐Cheol Baek, Eun‐Jeong Choi, Dong‐Hwan Shin, Hyoung‐Jae Kim, Haeyoun Choi, Hyoung‐Shik Shin, Dong‐Gyun Lim, Tai‐Gyu Kim

**Affiliations:** ^1^ Hematopoietic Stem Cell Bank, College of Medicine The Catholic University of Korea Seoul Korea; ^2^ Department of Microbiology, College of Medicine The Catholic University of Korea Seoul Korea; ^3^ Department of Infectious Diseases College of Medicine, Eulji University Daejeon Seogu Korea; ^4^ Translational Research Center, Research Institute of Public Health National Medical Center Seoul Korea

**Keywords:** HLA, MERS, MERS‐CoV, Middle East respiratory syndrome coronavirus

## Abstract

**Introduction:**

Middle East Respiratory Syndrome (MERS) caused by MERS‐coronavirus (CoV) is a lower respiratory tract disease characterized by a high mortality rate. MERS‐CoV spread from Saudi Arabia to other countries, including South Korea. Dysfunction of the human leukocyte antigen (HLA) system has many effects due to genetic complexity and its role in the adaptive immune response. We investigated the association of HLA class I and II alleles with MERS‐CoV in 32 patients with MERS.

**Methods:**

HLA‐A, ‐B, ‐C, ‐DRB1, ‐DQB1, and ‐DPB1 were genotyped by polymerase chain reaction sequence‐based typing.

**Results:**

HLA‐DQB1*03:02 are significantly associated with moderate/mild cases of MERS‐CoV. Other alleles are no statistical significance.

**Conclusions:**

Treatment strategies based on current research on the HLA gene and MERS‐CoV will provide potential therapeutic targets.

1

Middle East Respiratory Syndrome (MERS) is a lower respiratory tract disease reported in Saudi Arabia,^1^ and it has since spread to other countries. From 2013 to April 2018, a beta‐coronavirus of MERS infection (MERS‐CoV) was identified in dromedaries (*Camelus dromedarius*) in the field. Serological evidence in 20 countries and molecular evidence in 13 countries was found for viral circulation.^2^

South Korea is the most common location of MERS‐CoV infection outside the Arabian Peninsula by June 19, 2015. There is an approximately 21% risk of death in all cases of MERS (95% reliable interval: 14‐31).^3^ The interhospital spread began in one hospital and expanded to 17 hospitals in South Korea.^4^ The outbreak occurred entirely in hospitals and was primarily caused by infection control and policy failures. The average age of patients with MERS in South Korea was 55 years (global average: 50 years). Men were more affected than women in most countries, including South Korea. Most of the South Korean patients were infected at hospital and no community infections occurred. Respiratory disease, cancer, and hypertension were the main underlying diseases.^5^

The dysfunction of the human leukocyte antigen (HLA) system, the cause of many clinical disorders, has a wide range of effects due to genetic complexity as well as the role of adaptive immune response system.^6^ MERS‑CoV was investigated based on insights into the epidemiology and pathogenesis of the novel coronavirus of severe acute respiratory syndrome (SARS)‐CoV.^7^ Therefore, studies on the association of genetic variants including HLA with SARS or MERS has been ongoing.^8^ No HLA allele association was found in 95 Chinese patients with SARS.^9^ In the association study between HLA and SARS, HLA‐C*15:02 and DRB1*03:01 conferred resistance to SARS‐CoV infection (*p* < 0.05).^10^ HLA‐DRB1*12:02 of Vietnamese had association with SARS.^11^ HLA‑B*46:01 was associated with SARS in Taiwanese patients (*p* = 0.0008; *P*_c_ = 0.0279).^12^ In Taiwan, the odds ratio (OR) of SARS in people with the homozygous or heterozygous HLA‐C*08:01 genotype was 4.4 (95% confidence interval [CI], 1.5–12.9; *p* = 0.007).^13^ In another Taiwanese group, HLA‐B*46:01 was associated with the severity of SARS (*P*_c_ = 0.0279) after selecting only the "serious case" patient group compared with the high‐risk health‐care practitioner group in the infected "SARS latent."^12^ HLA‐B*07:03 frequencies in the SARS‐CoV group were significantly higher than those in the control group (OR = 4.08; 95% CI, 2.03–8.18; *p* = 0.00072; *P*_c_ < 0.0022), and the HLA‐DRB1*03:01 allele had a protective effect against SARS‐CoV infection (OR = 0.06; 95% CI, 0.01–0.47; *p* = 0.00008; *P*_c_ < 0.0042) in Chinese patients.^14^ In the Saudi Arabian population, HLA‐DRB1*11:01 and DQB1*02:02, HLA class II alleles, were associated with MERS‐CoV, but this was not related to the outcome of the disease.^15^ It is unclear whether the Korean case population is associated with a host genetic predisposition, such as HLA. In this study, we investigated the association of HLA class I and II alleles with MERS‐CoV.

This study comprised 32 patients with MERS admitted to or followed up after recovery at the National Medical Center in Seoul, South Korea. All patients were confirmed to have MERS‑CoV infection by real‑time reverse‐transcription polymerase chain reaction (Real Time RT‐PCR) of nasopharyngeal swabs or tracheal aspirates. The patients were classified into two groups depending on the clinical severity during their acute stage of infection. Severe disease (*n* = 16) included fatalities and patients who required mechanical ventilation to relieve respiratory failure. Moderate and mild disease (Mo/Mi) encompassed patients who reported symptoms such as fever, headache, cough, and malaise with or without pulmonary lesions in the absence of pulmonary failure. Demographic characteristics of the patients are shown in Table 1. Peripheral blood was collected from patients at the acute/convalescent phases of infection or after recovery. Peripheral blood mononuclear cells (PBMCs) were isolated from heparinized whole blood by density gradient centrifugation using Ficoll‐Paque solution (GE Healthcare) and stored in liquid nitrogen until use. The study was approved by the NMC Ethical Committee and written informed consent was obtained from the patients (institutional review board [IRB]: H‐1801‐086‐002, H‐1712‐085‐005).

**Table 1:** Characteristics of patients with MERS

	Group (clinical severity)		Total
	Moderate/mild	Severe	
No. of patients (%)	16 (50)	16 (50)	32
Male/female (*n*)	8/8	13/3	21/11
Age (year), mean ± *SD*	50 ± 21	58 ± 15	54 ± 19
Underlying disease, *n* (%)			
Diabetes	1 (6.3)	3 (18.8)	4 (12.5)
Chronic renal disease	1 (6.3)	2 (12.5)	3 (9.4)
Chronic cardiac disease	1 (6.3)	4 (25.0)	5 (15.6)
Chronic pulmonary disease	0 (0)	1 (6.3)	1 (3.1)
Hypertension	2 (12.5)	7 (43.8)	9 (28.1)
Chronic liver disease	1 (6.3)	0 (0)	1 (3.1)
Solid tumor	2 (12.5)	2 (12.5)	4 (12.5)
Obesity	0 (0)	1 (6.3)	1 (3.1)

Abbreviation: MERS, Middle East Respiratory Syndrome.

The control group included 142 genetically unrelated healthy Korean adults with no history of MERS and little or no gender distribution (age: 30–39 years; 70 females and 72 males). The control subjects provided written informed consent to participate and were mainly of comprised students and staff from the Medical College of the Catholic University of Korea and Hematopoietic Stem Cell Transplantation Center. The use of the material was reviewed and approved by the local institutional review board (IRB) of The Catholic University of Korea with written informed consent obtained for all samples collected (IRB: MC13SISI0126).

Genomic DNA was isolated from PBMCs by using the QIAamp DNA Blood Mini Kit (Qiagen) according to the manufacturer's instructions. DNA yields and purity were determined by measuring the absorbance at 260 and 280 nm with a spectrophotometer (Nanodrop 2000, Thermo Scientific).

The paired primers have previously been reported. PCR conditions and direct sequencing methods used were those described by Shin et al.^16^ Genotyping of HLA‐A, ‐B, ‐C, ‐DRB1, ‐DQB1, and ‐DPB1 was performed using PCR‐sequence‐based typing (PCR‐SBT) methods. PCR was performed as recommended by the 13th International Histocompatibility Workshop.

Statistical significance was determined using the *χ* ^2^ test and Fisher's exact test. We corrected the *p*‐values using Bonferroni's method and calculated the OR using Haldane's modification of Woolf's method. Each SNP was analyzed against the Hardy–Weinberg equilibrium of the control group using SNPStats (http://bioinfo.iconcologia.net/snpstats/start.htm) (*p* > 0.05).

HLA‐DQB1*03:02 showed a significant association with Mo/Mi cases of MERS‐CoV infection (OR = 4.8; 95% CI, 1.65–13.95; *p* = 0.002; *P*_c_ = 0.030). The HLA‐C*01:02 allele frequency decreased in the Mo/Mi cases compared with in the controls but did not show statistical significance by Bonferroni's correction (OR = 0.1; 95% CI, 0.017–1.018; *p* = 0.015; *P*_c_ = NS). In Mo/Mi cases of MERS‐CoV infection, the frequencies of HLA‐C*12:02, ‐DRB1*04:06, and ‐DRB1*14:03 alleles were higher than those in the controls, but were not statistically significant by Bonferroni's correction ([C*12:02] OR = 5.2; 95% CI, 1.17–23.40; *p* = 0.043; *P*_c_ = NS; [DRB1*04:06] OR = 5.4; 95% CI, 1.59–18.39; *p* = 0.011; *P*_c_ = NS; [DRB1*14:03] OR = 10.0; 95% CI, 1.31–76.56; *p* = 0.048; *P*_c_ = NS). In severe cases of MERS‐CoV infection, the frequencies of HLA‐C*07:06 and ‐DRB1*13:01 alleles were higher than those of the controls, but were not significant by Bonferroni's correction ([C*07:06] OR = 5.2; 95% CI, 1.17–23.40; *p* = 0.043; *P*_c_ = NS; [DRB1*13:01] OR = 10.0; 95% CI, 1.31–76.56; *p* = 0.048; *P*_c_ = NS). HLA‐DQB1*06:03 alleles carried statistical significance in the total cases (OR = 7.2; 95% CI, 1.16–45.29; *p* = 0.040; *P*_c_ = NS) and severe cases (OR = 10.0; 95% CI, 1.31–76.56; *p* = 0.048; *P*_c_ = NS); however, there was no significance by the correction (Table 2).

**Table 2:** Genetic influence of HLA class I and II in MERS patients

	Controls		MERS total		Moderate/mild		Severe	
Alleles	*n* = 142 (%)		*n* = 32 (%)		*n* = 16 (%)		*n* = 16 (%)	
C*01:02	48	(33.8)	7	(21.9)	1	(6.3)^b^^,^ ¥	6	(37.5)
C*07:06	6	(4.2)	4	(12.5)	1	(6.3)	3	(18.8)^g^^,^ ¥
C*12:02	6	(4.2)	3	(9.4)	3	(18.8)^c^^,^ ¥	0	(0.0)
DRB1*04:06	11	(7.7)	5	(15.6)	5	(31.3)^d^^,^ ¥	0	(0.0)
DRB1*13:01	2	(1.4)	3	(9.4)	1	(6.3)	2	(12.5)^h^^,^ ¥
DRB1*14:03	2	(1.4)	2	(6.3)	2	(12.5)^e^^,^ ¥	0	(0.0)
DQB1*03:02	30	(21.1)	10	(31.3)	9	(56.3)^f^	1	(6.3)
DQB1*06:03	2	(1.4)	3	(9.4)^a^^,^ ¥	1	(6.3)	2	(12.5)^i^^,^ ¥

Abbreviations: *P*_c_, Bonferroni's correction; MERS, Middle East Respiratory Syndrome; NS, not significant.

Controls versus total patients:

^a^ OR = 7.2 (1.16–45.29), *p* = 0.040, *P*_c_ = NS

Controls versus moderate/mild patients:

^b^ OR = 0.1 (0.017–1.018), *p* = 0.015, *P*_c_ = NS;

^c^ OR = 5.2 (1.17–23.40), *p* = 0.043, *P*_c_ = NS;

^d^ OR = 5.4 (1.59–18.39), *p* = 0.011, *P*_c_ = NS;

^e^ OR = 10.0 (1.31–76.56), *p* = 0.048, *P*_c_ = NS;

^f^ OR = 4.8 (1.65–13.95), *p* = 0.002, *P*_c_ = 0.030

Controls versus severe patients:

^g^ OR = 5.2 (1.17–23.40), *p* = 0.043, *P*_c_ = NS;

^h^ OR = 5.4 (1.59–18.39), *p* = 0.048, *P*_c_ = NS;

^i^ OR = 5.4 (1.59–18.39), *p* = 0.048, *P*_c_ = NS

^¥^ Fisher exact test

HLA‐A and ‐DPB1 alleles did not show statistical significance in the Korean cohorts compared with those of the controls. HLA‐B*11:01 allele frequency increased in the cases of the total and severe groups compared with the control group, but this was not statistically significant by Fisher's exact test (Table S1‐S6).

We investigated the association of HLA class I and II alleles with MERS‐CoV in 32 patients with MERS. HLA‐DQB1*03:02 showed a significant association with Mo/Mi cases of MERS‐CoV infection (OR = 4.8; 95% CI, 1.65–13.95; *p* = 0.002; *P*_c_ = 0.030). Cytokines play a pivotal role in aspirin‐exacerbated respiratory disease (AERD).^17^ These cytokines are secreted by the major histocompatibility complex class II, which is encoded by HLA class II genes (e.g., HLA‐DRB1, ‐DQB1, and ‐DPB1).^6^ According to pharmacogenetic tests for predicting the efficacy of aspirin desensitization (AD) in patients with respiratory disease exacerbated by aspirin, HLA‐DQB1*03:02 expression of nonresponders was significantly lower than that of respondents about AD (OR = 0.12; 95% CI, 0.02–0.76; *p* = 0.022). When predicting response to AD, HLA‐DQB1*03:02 had the sensitivity of 71.4% and the specificity of 81.8%.^17^ The HLA‐DQB1*03:02 allele expression in patients with AERD was higher (OR = 5.49; 95% CI, 2.40–12.59; *p* < 0.05). Haplotype expression of HLA‐DRB1*04/DQA1*03:01/DQB1*03:02 was higher (OR = 4.25; 95% CI, 1.94–9.29; *p* < 0.05).^18^ The HLA‐DQB1*03:02 allele is susceptible to AERD in patients.

Several studies have reported that HLA association with SARS‐CoV infection is closely related to that of MERS‐CoV.^9^, ^12^, ^19^ In Saudi Arabia, HLA‐DRB1*11:01 and DQB1*02:02 alleles were associated with MERS‐CoV infection.^15^ Conflicting results for HLA association with SARS‐CoV, which is closely related to MERS‐CoV, have been reported. While Xiong et al. and Ng et al.^9^, ^19^ reported no HLA associations, others have shown an association with HLA‑A*46:01.^12^

MERS‐CoV, SARS‐CoV, and SARS‐CoV‐2 are the members of the CoV family and belong to the β‐CoVs.^20^ Because MERS‐CoV mainly suppresses the innate immune system, stable transmission is possible in the early stages of infection. By limiting the production of interferons (IFNs) and recruiting neutrophils to the lungs, it can lead to robust production of proinflammatory cytokines and chemokines.^21^ A study of coronavirus infection‐19 (COVID‐19) found that the incidence of lymphopenia was 72%, consistent with SARS‐CoV and MERS‐CoV. CD4 ^+^ T cells, CD8 ^+^ T cells, and B cells were also reduced in 29%, 29%, and 25% of COVID‐19 patients, respectively. However, Natural Killer (NK) cell levels were considerably decreased in 59% of these cases (a higher incidence rate than that found for patients with MERS‐CoV or SARS‐CoV).^22^ This phenomenon has also been found in patients with MERS‐CoV or SARS‐CoV pneumonia.^23^ Lymphopenia occurs in 34% of patients with MERS. Lymphopenia can occur indirectly due to viral attachment of inflammatory mediators or immune disorders. Also, lymphopenia may develop when lymphocytes effuse into inflammatory lung tissue.^24^

To date, it is not clear why some patients suffer from MERS‐CoV. Treatment strategies based on current research on the HLA gene and its association with MERS‐CoV could provide potential therapeutic targets.

## CONFLICT OF INTERESTS

The authors declare that there are no conflict of interests.

## AUTHOR CONTRIBUTIONS

Baek In‐Cheol, Lim Dong‐Gyun, and Kim Tai‐Gyu contributed to the conception of the study; Baek In‐Cheol was involved in data acquisition and formal analysis; Baek In‐Cheol, Lim Dong‐Gyun, and Kim Tai‐Gyu were involved in funding acquisition; Baek In‐Cheol, Choi Eun‐Jeong, Shin Dong‐Hwan, and Kim Hyoung‐Jae performed the investigation and methodology; Lim Dong‐Gyun and Kim Tai‐Gyu were involved in project administration; Shin Hyoung‐Shik and Lim Dong‐Gyun acquired resources; Kim Tai‐Gyu provided supervision; Baek In‐Cheol, Shin Dong‐Hwan, Kim Hyoung‐Jae, and Choi Haeyoun were involved in validation; Baek In‐Cheol performed statistical analysis; Baek In‐Cheol wrote the original draft, as well as reviewed and edited the manuscript.

## Supporting information

Supplementary information.

## Data Availability

The data that support the findings of this study are available on request from the corresponding author. The data are not publicly available due to privacy or ethical restrictions.
