# Supplementary material for: Association of HLA class I and II genes with Middle East respiratory syndrome coronavirus infection in Koreans
Source: Immun Inflamm Dis. 2021 Oct 12;10(1):111–6. doi: 10.1002/iid3.541 (PMC8669699; doi:10.1002/iid3.541)
Supplement: Supplementary file 1 — Supplementary information. [file IID3-10-111-s001.docx]

| **Table S1.** Genetic influence of HLA-A in MERS patients | | | | | | | | | | |  |  |  |  |  |  |  |  |  |  |  |  |  |  |  |  |  |
| --- | --- | --- | --- | --- | --- | --- | --- | --- | --- | --- | --- | --- | --- | --- | --- | --- | --- | --- | --- | --- | --- | --- | --- | --- | --- | --- | --- |
|  |  |  |  | Controls | |  | MERS total | | | | | |  | Moderate/Mild cases | | | | | |  | Severe cases | | | | | |  |
|  | Locus | Alleles |  | n = 142 (%) | |  | n = 32 (%) | | χ^2^ | p-value | P_c_ | OR |  | n = 16 (%) | | χ^2^ | p-value | P_c_ | OR |  | n = 16 (%) | | χ^2^ | p-value | P_c_ | OR |  |
|  | A | 01:01 |  | 4 | (2.8) |  | 2 | (6.3) | 0.924 | 0.336 | 6.053 | NA |  | 1 | (6.3) | 0.553 | 0.457 | 8.227 | NA |  | 1 | (6.3) | 0.553 | 0.457 | 8.227 | NA |  |
|  | A | 02:01 |  | 53 | (37.3) |  | 8 | (25.0) | 1.742 | 0.187 | 3.364 | NA |  | 4 | (25.0) | 0.947 | 0.330 | 5.949 | NA |  | 4 | (25.0) | 0.947 | 0.330 | 5.949 | NA |  |
|  | A | 02:03 |  | 5 | (3.5) |  | 0 | (0.0) | 1.160 | 0.281 | 5.066 | NA |  | 0 | (0.0) | 0.582 | 0.446 | 8.021 | NA |  | 0 | (0.0) | 0.582 | 0.446 | 8.021 | NA |  |
|  | A | 02:06 |  | 24 | (16.9) |  | 5 | (15.6) | 0.031 | 0.861 | 15.499 | NA |  | 3 | (18.8) | 0.035 | 0.852 | 15.341 | NA |  | 2 | (12.5) | 0.203 | 0.653 | 11.747 | NA |  |
|  | A | 02:07 |  | 8 | (5.6) |  | 3 | (9.4) | 0.617 | 0.432 | 7.778 | NA |  | 1 | (6.3) | 0.010 | 0.920 | 16.555 | NA |  | 2 | (12.5) | 1.144 | 0.285 | 5.128 | NA |  |
|  | A | 03:01 |  | 2 | (1.4) |  | 1 | (3.1) | 0.454 | 0.500 | 9.007 | NA |  | 0 | (0.0) | 0.228 | 0.633 | 11.391 | NA |  | 1 | (6.3) | 1.810 | 0.179 | 3.214 | NA |  |
|  | A | 11:01 |  | 33 | (23.2) |  | 5 | (15.6) | 0.887 | 0.346 | 6.233 | NA |  | 3 | (18.8) | 0.165 | 0.685 | 12.327 | NA |  | 2 | (12.5) | 0.962 | 0.327 | 5.882 | NA |  |
|  | A | 11:02 |  | 1 | (0.7) |  | 1 | (3.1) | 1.347 | 0.246 | 4.425 | NA |  | 1 | (6.3) | 3.539 | 0.060 | 1.079 | NA |  | 0 | (0.0) | 0.113 | 0.736 | 13.254 | NA |  |
|  | A | 24:02 |  | 46 | (32.4) |  | 14 | (43.8) | 1.491 | 0.222 | 3.998 | NA |  | 7 | (43.8) | 0.832 | 0.362 | 6.512 | NA |  | 7 | (43.8) | 0.832 | 0.362 | 6.512 | NA |  |
|  | A | 26:01 |  | 9 | (6.3) |  | 2 | (6.3) | 0.000 | 0.985 | 17.735 | NA |  | 2 | (12.5) | 0.843 | 0.359 | 6.454 | NA |  | 0 | (0.0) | 1.075 | 0.300 | 5.395 | NA |  |
|  | A | 26:02 |  | 15 | (10.6) |  | 0 | (0.0) | 3.699 | 0.054 | 0.980 | NA |  | 0 | (0.0) | 1.867 | 0.172 | 3.092 | NA |  | 0 | (0.0) | 1.867 | 0.172 | 3.092 | NA |  |
|  | A | 26:03 |  | 1 | (0.7) |  | 0 | (0.0) | 0.227 | 0.634 | 11.412 | NA |  | 0 | (0.0) | 0.113 | 0.736 | 13.254 | NA |  | 0 | (0.0) | 0.113 | 0.736 | 13.254 | NA |  |
|  | A | 29:01 |  | 1 | (0.7) |  | 1 | (3.1) | 1.347 | 0.246 | 4.425 | NA |  | 1 | (6.3) | 3.539 | 0.060 | 1.079 | NA |  | 0 | (0.0) | 0.113 | 0.736 | 13.254 | NA |  |
|  | A | 30:01 |  | 12 | (8.5) |  | 4 | (12.5) | 0.513 | 0.474 | 8.531 | NA |  | 1 | (6.3) | 0.092 | 0.761 | 13.704 | NA |  | 3 | (18.8) | 1.775 | 0.183 | 3.289 | NA |  |
|  | A | 30:04 |  | 9 | (6.3) |  | 0 | (0.0) | 2.139 | 0.144 | 2.585 | NA |  | 0 | (0.0) | 1.075 | 0.300 | 5.395 | NA |  | 0 | (0.0) | 1.075 | 0.300 | 5.395 | NA |  |
|  | A | 31:01 |  | 11 | (7.7) |  | 4 | (12.5) | 0.749 | 0.387 | 6.962 | NA |  | 1 | (6.3) | 0.046 | 0.830 | 14.947 | NA |  | 3 | (18.8) | 2.156 | 0.142 | 2.556 | NA |  |
|  | A | 32:01 |  | 1 | (0.7) |  | 0 | (0.0) | 0.227 | 0.634 | 11.412 | NA |  | 0 | (0.0) | 0.113 | 0.736 | 13.254 | NA |  | 0 | (0.0) | 0.113 | 0.736 | 13.254 | NA |  |
|  | A | 33:03 |  | 38 | (26.8) |  | 8 | (25.0) | 0.042 | 0.838 | 15.090 | NA |  | 5 | (31.3) | 0.146 | 0.702 | 12.638 | NA |  | 3 | (18.8) | 0.480 | 0.488 | 8.790 | NA |  |

P_c_, Bonferroni's correction; NA, not applicable

| **Table S2.** Genetic influence of HLA-B in MERS patients | | | | | | | | | | |  |  |  |  |  |  |  |  |  |  |  |  |  |  |  |  |  |
| --- | --- | --- | --- | --- | --- | --- | --- | --- | --- | --- | --- | --- | --- | --- | --- | --- | --- | --- | --- | --- | --- | --- | --- | --- | --- | --- | --- |
|  |  |  |  | Controls | |  | MERS total | | | | | |  | Moderate/Mild cases | | | | | |  | Severe cases | | | | | |  |
|  | Locus | Alleles |  | n = 142 (%) | |  | n = 32 (%) | | χ^2^ | p-value | P_c_ | OR |  | n = 16 (%) | | χ^2^ | p-value | P_c_ | OR |  | n = 16 (%) | | χ^2^ | p-value | P_c_ | OR |  |
|  | B | 07:02 |  | 13 | (9.2) |  | 2 | (6.3) | 0.280 | 0.597 | 20.890 | NA |  | 1 | (6.3) | 0.150 | 0.698 | 24.440 | NA |  | 1 | (6.3) | 0.150 | 0.698 | 24.440 | NA |  |
|  | B | 07:05 |  | 2 | (1.4) |  | 1 | (3.1) | 0.454 | 0.500 | 17.513 | NA |  | 1 | (6.3) | 1.810 | 0.179 | 6.249 | NA |  | 0 | (0.0) | 0.228 | 0.633 | 22.149 | NA |  |
|  | B | 08:01 |  | 2 | (1.4) |  | 0 | (0.0) | 0.456 | 0.500 | 17.483 | NA |  | 0 | (0.0) | 0.228 | 0.633 | 22.149 | NA |  | 0 | (0.0) | 0.228 | 0.633 | 22.149 | NA |  |
|  | B | 13:01 |  | 4 | (2.8) |  | 1 | (3.1) | 0.009 | 0.925 | 32.372 | NA |  | 1 | (6.3) | 0.553 | 0.457 | 15.997 | NA |  | 0 | (0.0) | 0.462 | 0.496 | 17.377 | NA |  |
|  | B | 13:02 |  | 13 | (9.2) |  | 2 | (6.3) | 0.280 | 0.597 | 20.890 | NA |  | 0 | (0.0) | 1.596 | 0.206 | 7.226 | NA |  | 2 | (12.5) | 0.187 | 0.665 | 23.282 | NA |  |
|  | B | 14:01 |  | 7 | (4.9) |  | 0 | (0.0) | 1.644 | 0.200 | 6.994 | NA |  | 0 | (0.0) | 0.825 | 0.364 | 12.727 | NA |  | 0 | (0.0) | 0.825 | 0.364 | 12.727 | NA |  |
|  | B | 15:01 |  | 22 | (15.5) |  | 4 | (12.5) | 0.184 | 0.668 | 23.377 | NA |  | 3 | (18.8) | 0.115 | 0.735 | 25.727 | NA |  | 1 | (6.3) | 0.988 | 0.320 | 11.211 | NA |  |
|  | B | 15:07 |  | 3 | (2.1) |  | 1 | (3.1) | 0.119 | 0.730 | 25.548 | NA |  | 0 | (0.0) | 0.345 | 0.557 | 19.502 | NA |  | 1 | (6.3) | 0.998 | 0.318 | 11.127 | NA |  |
|  | B | 15:11 |  | 3 | (2.1) |  | 3 | (9.4) | 4.137 | 0.066^¥^ | 2.296 | NA |  | 1 | (6.3) | 0.998 | 0.318 | 11.127 | NA |  | 2 | (12.5) | 5.063 | 0.073^¥^ | 25.495 | NA |  |
|  | B | 15:18 |  | 5 | (3.5) |  | 0 | (0.0) | 1.160 | 0.281 | 9.851 | NA |  | 0 | (0.0) | 0.582 | 0.446 | 15.596 | NA |  | 0 | (0.0) | 0.582 | 0.446 | 15.596 | NA |  |
|  | B | 27:04 |  | 1 | (0.7) |  | 1 | (3.1) | 1.347 | 0.246 | 8.604 | NA |  | 1 | (6.3) | 3.539 | 0.060 | 2.098 | NA |  | 0 | (0.0) | 0.113 | 0.736 | 25.771 | NA |  |
|  | B | 27:05 |  | 10 | (7.0) |  | 0 | (0.0) | 2.391 | 0.122 | 4.271 | NA |  | 0 | (0.0) | 1.203 | 0.273 | 9.546 | NA |  | 0 | (0.0) | 1.203 | 0.273 | 9.546 | NA |  |
|  | B | 35:01 |  | 15 | (10.6) |  | 3 | (9.4) | 0.040 | 0.842 | 29.468 | NA |  | 2 | (12.5) | 0.056 | 0.813 | 28.443 | NA |  | 1 | (6.3) | 0.294 | 0.588 | 20.569 | NA |  |
|  | B | 35:03 |  | 1 | (0.7) |  | 0 | (0.0) | 0.227 | 0.634 | 22.191 | NA |  | 0 | (0.0) | 0.113 | 0.736 | 25.771 | NA |  | 0 | (0.0) | 0.113 | 0.736 | 25.771 | NA |  |
|  | B | 37:01 |  | 5 | (3.5) |  | 2 | (6.3) | 0.504 | 0.478 | 16.726 | NA |  | 1 | (6.3) | 0.293 | 0.588 | 20.588 | NA |  | 1 | (6.3) | 0.293 | 0.588 | 20.588 | NA |  |
|  | B | 38:02 |  | 6 | (4.2) |  | 1 | (3.1) | 0.082 | 0.775 | 27.116 | NA |  | 1 | (6.3) | 0.139 | 0.709 | 24.817 | NA |  | 0 | (0.0) | 0.703 | 0.402 | 14.065 | NA |  |
|  | B | 39:01 |  | 2 | (1.4) |  | 1 | (3.1) | 0.454 | 0.500 | 17.513 | NA |  | 1 | (6.3) | 1.810 | 0.179 | 6.249 | NA |  | 0 | (0.0) | 0.228 | 0.633 | 22.149 | NA |  |
|  | B | 40:01 |  | 10 | (7.0) |  | 2 | (6.3) | 0.026 | 0.873 | 30.557 | NA |  | 1 | (6.3) | 0.014 | 0.906 | 31.711 | NA |  | 1 | (6.3) | 0.014 | 0.906 | 31.711 | NA |  |
|  | B | 40:02 |  | 9 | (6.3) |  | 4 | (12.5) | 1.434 | 0.231 | 8.087 | NA |  | 3 | (18.8) | 3.157 | 0.076 | 2.647 | NA |  | 1 | (6.3) | 0.000 | 0.989 | 34.617 | NA |  |
|  | B | 40:03 |  | 2 | (1.4) |  | 1 | (3.1) | 0.454 | 0.500 | 17.513 | NA |  | 1 | (6.3) | 1.810 | 0.179 | 6.249 | NA |  | 0 | (0.0) | 0.228 | 0.633 | 22.149 | NA |  |
|  | B | 40:06 |  | 10 | (7.0) |  | 3 | (9.4) | 0.206 | 0.650 | 22.759 | NA |  | 2 | (12.5) | 0.610 | 0.435 | 15.213 | NA |  | 1 | (6.3) | 0.014 | 0.906 | 31.711 | NA |  |
|  | B | 44:02 |  | 3 | (2.1) |  | 1 | (3.1) | 0.119 | 0.730 | 25.548 | NA |  | 0 | (0.0) | 0.345 | 0.557 | 19.502 | NA |  | 1 | (6.3) | 0.998 | 0.318 | 11.127 | NA |  |
|  | B | 44:03 |  | 19 | (13.4) |  | 7 | (21.9) | 1.483 | 0.223 | 7.817 | NA |  | 3 | (18.8) | 0.346 | 0.556 | 19.475 | NA |  | 4 | (25.0) | 1.561 | 0.212 | 7.403 | NA |  |
|  | B | 46:01 |  | 16 | (11.3) |  | 4 | (12.5) | 0.039 | 0.843 | 29.522 | NA |  | 1 | (6.3) | 0.377 | 0.539 | 18.872 | NA |  | 3 | (18.8) | 0.761 | 0.383 | 13.406 | NA |  |
|  | B | 48:01 |  | 15 | (10.6) |  | 3 | (9.4) | 0.040 | 0.842 | 29.468 | NA |  | 1 | (6.3) | 0.294 | 0.588 | 20.569 | NA |  | 2 | (12.5) | 0.056 | 0.813 | 28.443 | NA |  |
|  | B | 51:01 |  | 24 | (16.9) |  | 5 | (15.6) | 0.031 | 0.861 | 30.137 | NA |  | 2 | (12.5) | 0.203 | 0.653 | 22.841 | NA |  | 3 | (18.8) | 0.035 | 0.852 | 29.829 | NA |  |
|  | B | 51:02 |  | 3 | (2.1) |  | 0 | (0.0) | 0.688 | 0.407 | 14.241 | NA |  | 0 | (0.0) | 0.345 | 0.557 | 19.502 | NA |  | 0 | (0.0) | 0.345 | 0.557 | 19.502 | NA |  |
|  | B | 52:01 |  | 5 | (3.5) |  | 2 | (6.3) | 0.504 | 0.478 | 16.726 | NA |  | 2 | (12.5) | 2.738 | 0.098 | 3.430 | NA |  | 0 | (0.0) | 0.582 | 0.446 | 15.596 | NA |  |
|  | B | 54:01 |  | 20 | (14.1) |  | 2 | (6.3) | 1.451 | 0.228 | 7.991 | NA |  | 1 | (6.3) | 0.766 | 0.382 | 13.353 | NA |  | 1 | (6.3) | 0.766 | 0.382 | 13.353 | NA |  |
|  | B | 55:02 |  | 6 | (4.2) |  | 1 | (3.1) | 0.082 | 0.775 | 27.116 | NA |  | 0 | (0.0) | 0.703 | 0.402 | 14.065 | NA |  | 1 | (6.3) | 0.139 | 0.709 | 24.817 | NA |  |
|  | B | 56:01 |  | 1 | (0.7) |  | 0 | (0.0) | 0.227 | 0.634 | 22.191 | NA |  | 0 | (0.0) | 0.113 | 0.736 | 25.771 | NA |  | 0 | (0.0) | 0.113 | 0.736 | 25.771 | NA |  |
|  | B | 57:01 |  | 1 | (0.7) |  | 0 | (0.0) | 0.227 | 0.634 | 22.191 | NA |  | 0 | (0.0) | 0.113 | 0.736 | 25.771 | NA |  | 0 | (0.0) | 0.113 | 0.736 | 25.771 | NA |  |
|  | B | 58:01 |  | 16 | (11.3) |  | 1 | (3.1) | 1.964 | 0.161 | 5.638 | NA |  | 1 | (6.3) | 0.377 | 0.539 | 18.872 | NA |  | 0 | (0.0) | 2.006 | 0.157 | 5.484 | NA |  |
|  | B | 59:01 |  | 3 | (2.1) |  | 1 | (3.1) | 0.119 | 0.730 | 25.548 | NA |  | 0 | (0.0) | 0.345 | 0.557 | 19.502 | NA |  | 1 | (6.3) | 0.998 | 0.318 | 11.127 | NA |  |
|  | B | 67:01 |  | 4 | (2.8) |  | 0 | (0.0) | 0.923 | 0.337 | 11.788 | NA |  | 0 | (0.0) | 0.462 | 0.496 | 17.377 | NA |  | 0 | (0.0) | 0.462 | 0.496 | 17.377 | NA |  |

P_c_, Bonferroni's correction; NA, not applicable; ¥, Fisher exact test

| **Table S3.** Genetic influence of HLA-C in MERS patients | | | | | | | | | | |  |  |  |  |  |  |  |  |  |  |  |  |  |  |  |  |  |
| --- | --- | --- | --- | --- | --- | --- | --- | --- | --- | --- | --- | --- | --- | --- | --- | --- | --- | --- | --- | --- | --- | --- | --- | --- | --- | --- | --- |
|  |  |  |  | Controls | |  | MERS total | | | | | |  | Moderate/Mild cases | | | | | |  | Severe cases | | | | | |  |
|  | Locus | Alleles |  | n = 142 (%) | |  | n = 32 (%) | | χ^2^ | p-value | P_c_ | OR |  | n = 16 (%) | | χ^2^ | p-value | P_c_ | OR |  | n = 16 (%) | | χ^2^ | p-value | P_c_ | OR |  |
|  | C1 | 01:02 |  | 48 | (33.8) |  | 7 | (21.9) | 1.719 | 0.190 | 3.987 | NA |  | 1 | (6.3) | 5.102 | **0.015^¥^** | 0.318 | **0.1** |  | 6 | (37.5) | 0.087 | 0.768 | 16.118 | NA |  |
|  | C1 | 01:03 |  | 3 | (2.1) |  | 1 | (3.1) | 0.119 | 0.730 | 15.329 | NA |  | 0 | (0.0) | 0.345 | 0.557 | 11.701 | NA |  | 1 | (6.3) | 0.998 | 0.318 | 6.676 | NA |  |
|  | C2 | 02:02 |  | 1 | (0.7) |  | 0 | (0.0) | 0.227 | 0.634 | 13.314 | NA |  | 0 | (0.0) | 0.113 | 0.736 | 15.463 | NA |  | 0 | (0.0) | 0.113 | 0.736 | 15.463 | NA |  |
|  | C1 | 03:02 |  | 16 | (11.3) |  | 1 | (3.1) | 1.964 | 0.161 | 3.383 | NA |  | 1 | (6.3) | 0.377 | 0.539 | 11.323 | NA |  | 0 | (0.0) | 2.006 | 0.157 | 3.290 | NA |  |
|  | C1 | 03:03 |  | 28 | (19.7) |  | 7 | (21.9) | 0.076 | 0.783 | 16.451 | NA |  | 3 | (18.8) | 0.009 | 0.926 | 19.453 | NA |  | 4 | (25.0) | 0.248 | 0.618 | 12.983 | NA |  |
|  | C1 | 03:04 |  | 21 | (14.8) |  | 6 | (18.8) | 0.313 | 0.576 | 12.098 | NA |  | 5 | (31.3) | 2.834 | 0.092 | 1.938 | NA |  | 1 | (6.3) | 0.875 | 0.350 | 7.342 | NA |  |
|  | C2 | 04:01 |  | 14 | (9.9) |  | 3 | (9.4) | 0.007 | 0.934 | 19.605 | NA |  | 3 | (18.8) | 1.184 | 0.277 | 5.808 | NA |  | 0 | (0.0) | 1.731 | 0.188 | 3.954 | NA |  |
|  | C2 | 05:01 |  | 3 | (2.1) |  | 1 | (3.1) | 0.119 | 0.730 | 15.329 | NA |  | 0 | (0.0) | 0.345 | 0.557 | 11.701 | NA |  | 1 | (6.3) | 0.998 | 0.318 | 6.676 | NA |  |
|  | C2 | 06:02 |  | 18 | (12.7) |  | 6 | (18.8) | 0.810 | 0.368 | 7.729 | NA |  | 2 | (12.5) | 0.000 | 0.984 | 20.664 | NA |  | 4 | (25.0) | 1.822 | 0.177 | 3.718 | NA |  |
|  | C1 | 07:02 |  | 25 | (17.6) |  | 3 | (9.4) | 1.310 | 0.252 | 5.299 | NA |  | 2 | (12.5) | 0.265 | 0.607 | 12.747 | NA |  | 1 | (6.3) | 1.349 | 0.245 | 5.155 | NA |  |
|  | C1 | 07:04 |  | 5 | (3.5) |  | 0 | (0.0) | 1.160 | 0.281 | 5.910 | NA |  | 0 | (0.0) | 0.582 | 0.446 | 9.358 | NA |  | 0 | (0.0) | 0.582 | 0.446 | 9.358 | NA |  |
|  | C1 | 07:06 |  | 6 | (4.2) |  | 4 | (12.5) | 3.301 | 0.069 | 1.454 | NA |  | 1 | (6.3) | 0.139 | 0.709 | 14.890 | NA |  | 3 | (18.8) | 5.647 | **0.043^¥^** | 8.973 | **5.2** |  |
|  | C1 | 08:01 |  | 22 | (15.5) |  | 7 | (21.9) | 0.766 | 0.382 | 8.012 | NA |  | 4 | (25.0) | 0.945 | 0.331 | 6.949 | NA |  | 3 | (18.8) | 0.115 | 0.735 | 15.436 | NA |  |
|  | C1 | 08:02 |  | 7 | (4.9) |  | 0 | (0.0) | 1.644 | 0.200 | 4.197 | NA |  | 0 | (0.0) | 0.825 | 0.364 | 7.636 | NA |  | 0 | (0.0) | 0.825 | 0.364 | 7.636 | NA |  |
|  | C1 | 08:03 |  | 3 | (2.1) |  | 0 | (0.0) | 0.688 | 0.407 | 8.544 | NA |  | 0 | (0.0) | 0.345 | 0.557 | 11.701 | NA |  | 0 | (0.0) | 0.345 | 0.557 | 11.701 | NA |  |
|  | C1 | 12:02 |  | 6 | (4.2) |  | 3 | (9.4) | 1.412 | 0.235 | 4.929 | NA |  | 3 | (18.8) | 5.647 | **0.043^¥^** | 8.973 | **5.2** |  | 0 | (0.0) | 0.703 | 0.402 | 8.439 | NA |  |
|  | C1 | 12:03 |  | 2 | (1.4) |  | 0 | (0.0) | 0.456 | 0.500 | 10.490 | NA |  | 0 | (0.0) | 0.228 | 0.633 | 13.289 | NA |  | 0 | (0.0) | 0.228 | 0.633 | 13.289 | NA |  |
|  | C1 | 14:02 |  | 21 | (14.8) |  | 5 | (15.6) | 0.014 | 0.905 | 18.996 | NA |  | 2 | (12.5) | 0.061 | 0.806 | 16.918 | NA |  | 3 | (18.8) | 0.175 | 0.676 | 14.187 | NA |  |
|  | C1 | 14:03 |  | 15 | (10.6) |  | 3 | (9.4) | 0.040 | 0.842 | 17.681 | NA |  | 2 | (12.5) | 0.056 | 0.813 | 17.066 | NA |  | 1 | (6.3) | 0.294 | 0.588 | 12.342 | NA |  |
|  | C2 | 15:02 |  | 8 | (5.6) |  | 1 | (3.1) | 0.335 | 0.563 | 11.816 | NA |  | 0 | (0.0) | 0.949 | 0.330 | 6.927 | NA |  | 1 | (6.3) | 0.010 | 0.920 | 19.314 | NA |  |
|  | C2 | 15:05 |  | 1 | (0.7) |  | 1 | (3.1) | 1.347 | 0.246 | 5.162 | NA |  | 0 | (0.0) | 0.113 | 0.736 | 15.463 | NA |  | 1 | (6.3) | 3.539 | 0.060 | 1.259 | NA |  |

P_c_, Bonferroni's correction; NA, not applicable; ¥, Fisher exact test

| **Table S4.** Genetic influence of HLA-DRB1 in MERS patients | | | | | | | | | | |  |  |  |  |  |  |  |  |  |  |  |  |  |  |  |  |  |
| --- | --- | --- | --- | --- | --- | --- | --- | --- | --- | --- | --- | --- | --- | --- | --- | --- | --- | --- | --- | --- | --- | --- | --- | --- | --- | --- | --- |
|  |  |  |  | Controls | |  | MERS total | | | | | |  | Moderate/Mild cases | | | | | |  | Severe cases | | | | | |  |
|  | Locus | Alleles |  | n = 142 (%) | |  | n = 32 (%) | | χ^2^ | p-value | P_c_ | OR |  | n = 16 (%) | | χ^2^ | p-value | P_c_ | OR |  | n = 16 (%) | | χ^2^ | p-value | P_c_ | OR |  |
|  | DRB1 | 01:01 |  | 20 | (14.1) |  | 1 | (3.1) | 2.956 | 0.086 | 2.396 | NA |  | 0 | (0.0) | 2.580 | 0.108 | 3.030 | NA |  | 1 | (6.3) | 0.766 | 0.382 | 10.682 | NA |  |
|  | DRB1 | 03:01 |  | 8 | (5.6) |  | 0 | (0.0) | 1.890 | 0.169 | 4.739 | NA |  | 0 | (0.0) | 0.949 | 0.330 | 9.236 | NA |  | 0 | (0.0) | 0.949 | 0.330 | 9.236 | NA |  |
|  | DRB1 | 04:01 |  | 2 | (1.4) |  | 0 | (0.0) | 0.456 | 0.500 | 13.987 | NA |  | 0 | (0.0) | 0.228 | 0.633 | 17.719 | NA |  | 0 | (0.0) | 0.228 | 0.633 | 17.719 | NA |  |
|  | DRB1 | 04:03 |  | 8 | (5.6) |  | 3 | (9.4) | 0.617 | 0.432 | 12.098 | NA |  | 2 | (12.5) | 1.144 | 0.285 | 7.978 | NA |  | 1 | (6.3) | 0.010 | 0.920 | 25.751 | NA |  |
|  | DRB1 | 04:04 |  | 6 | (4.2) |  | 0 | (0.0) | 1.400 | 0.237 | 6.626 | NA |  | 0 | (0.0) | 0.703 | 0.402 | 11.252 | NA |  | 0 | (0.0) | 0.703 | 0.402 | 11.252 | NA |  |
|  | DRB1 | 04:05 |  | 16 | (11.3) |  | 4 | (12.5) | 0.039 | 0.843 | 23.617 | NA |  | 2 | (12.5) | 0.022 | 0.883 | 24.726 | NA |  | 2 | (12.5) | 0.022 | 0.883 | 24.726 | NA |  |
|  | DRB1 | 04:06 |  | 11 | (7.7) |  | 5 | (15.6) | 1.941 | 0.164 | 4.579 | NA |  | 5 | (31.3) | 8.728 | **0.011^¥^** | 0.297 | **5.4** |  | 0 | (0.0) | 1.332 | 0.248 | 6.956 | NA |  |
|  | DRB1 | 04:07 |  | 3 | (2.1) |  | 1 | (3.1) | 0.119 | 0.730 | 20.439 | NA |  | 0 | (0.0) | 0.345 | 0.557 | 15.602 | NA |  | 1 | (6.3) | 0.998 | 0.318 | 8.901 | NA |  |
|  | DRB1 | 04:10 |  | 3 | (2.1) |  | 0 | (0.0) | 0.688 | 0.407 | 11.392 | NA |  | 0 | (0.0) | 0.345 | 0.557 | 15.602 | NA |  | 0 | (0.0) | 0.345 | 0.557 | 15.602 | NA |  |
|  | DRB1 | 07:01 |  | 19 | (13.4) |  | 5 | (15.6) | 0.111 | 0.739 | 20.703 | NA |  | 1 | (6.3) | 0.661 | 0.416 | 11.651 | NA |  | 4 | (25.0) | 1.561 | 0.212 | 5.923 | NA |  |
|  | DRB1 | 08:02 |  | 10 | (7.0) |  | 3 | (9.4) | 0.206 | 0.650 | 18.207 | NA |  | 2 | (12.5) | 0.610 | 0.435 | 12.171 | NA |  | 1 | (6.3) | 0.014 | 0.906 | 25.369 | NA |  |
|  | DRB1 | 08:03 |  | 20 | (14.1) |  | 8 | (25.0) | 2.304 | 0.129 | 3.612 | NA |  | 3 | (18.8) | 0.252 | 0.616 | 17.246 | NA |  | 5 | (31.3) | 3.181 | 0.074 | 2.086 | NA |  |
|  | DRB1 | 09:01 |  | 23 | (16.2) |  | 5 | (15.6) | 0.006 | 0.937 | 26.224 | NA |  | 2 | (12.5) | 0.148 | 0.701 | 19.624 | NA |  | 3 | (18.8) | 0.068 | 0.794 | 22.233 | NA |  |
|  | DRB1 | 10:01 |  | 4 | (2.8) |  | 2 | (6.3) | 0.924 | 0.336 | 9.416 | NA |  | 1 | (6.3) | 0.553 | 0.457 | 12.798 | NA |  | 1 | (6.3) | 0.553 | 0.457 | 12.798 | NA |  |
|  | DRB1 | 11:01 |  | 12 | (8.5) |  | 0 | (0.0) | 2.905 | 0.088 | 2.473 | NA |  | 0 | (0.0) | 1.463 | 0.226 | 6.340 | NA |  | 0 | (0.0) | 1.463 | 0.226 | 6.340 | NA |  |
|  | DRB1 | 11:06 |  | 1 | (0.7) |  | 0 | (0.0) | 0.227 | 0.634 | 17.752 | NA |  | 0 | (0.0) | 0.113 | 0.736 | 20.617 | NA |  | 0 | (0.0) | 0.113 | 0.736 | 20.617 | NA |  |
|  | DRB1 | 12:01 |  | 9 | (6.3) |  | 3 | (9.4) | 0.375 | 0.540 | 15.126 | NA |  | 0 | (0.0) | 1.075 | 0.300 | 8.393 | NA |  | 3 | (18.8) | 3.157 | 0.076 | 2.117 | NA |  |
|  | DRB1 | 12:02 |  | 10 | (7.0) |  | 4 | (12.5) | 1.051 | 0.305 | 8.545 | NA |  | 1 | (6.3) | 0.014 | 0.906 | 25.369 | NA |  | 3 | (18.8) | 2.610 | 0.106 | 2.973 | NA |  |
|  | DRB1 | 13:01 |  | 2 | (1.4) |  | 3 | (9.4) | 5.938 | 0.069^¥^ | 1.934 | NA |  | 1 | (6.3) | 1.810 | 0.179 | 5.000 | NA |  | 2 | (12.5) | 7.169 | **0.048^¥^** | 1.346 | **###** |  |
|  | DRB1 | 13:02 |  | 23 | (16.2) |  | 5 | (15.6) | 0.006 | 0.937 | 26.224 | NA |  | 3 | (18.8) | 0.068 | 0.794 | 22.233 | NA |  | 2 | (12.5) | 0.148 | 0.701 | 19.624 | NA |  |
|  | DRB1 | 14:03 |  | 2 | (1.4) |  | 2 | (6.3) | 2.726 | 0.099 | 2.765 | NA |  | 2 | (12.5) | 7.169 | **0.048^¥^** | 1.346 | **###** |  | 0 | (0.0) | 0.228 | 0.633 | 17.719 | NA |  |
|  | DRB1 | 14:04 |  | 1 | (0.7) |  | 0 | (0.0) | 0.227 | 0.634 | 17.752 | NA |  | 0 | (0.0) | 0.113 | 0.736 | 20.617 | NA |  | 0 | (0.0) | 0.113 | 0.736 | 20.617 | NA |  |
|  | DRB1 | 14:05 |  | 15 | (10.6) |  | 2 | (6.3) | 0.551 | 0.458 | 12.820 | NA |  | 2 | (12.5) | 0.056 | 0.813 | 22.754 | NA |  | 0 | (0.0) | 1.867 | 0.172 | 4.810 | NA |  |
|  | DRB1 | 14:06 |  | 4 | (2.8) |  | 0 | (0.0) | 0.923 | 0.337 | 9.430 | NA |  | 0 | (0.0) | 0.462 | 0.496 | 13.902 | NA |  | 0 | (0.0) | 0.462 | 0.496 | 13.902 | NA |  |
|  | DRB1 | 14:54 |  | 6 | (4.2) |  | 2 | (6.3) | 0.244 | 0.621 | 17.396 | NA |  | 1 | (6.3) | 0.139 | 0.709 | 19.854 | NA |  | 1 | (6.3) | 0.139 | 0.709 | 19.854 | NA |  |
|  | DRB1 | 15:01 |  | 21 | (14.8) |  | 2 | (6.3) | 1.660 | 0.198 | 5.533 | NA |  | 1 | (6.3) | 0.875 | 0.350 | 9.790 | NA |  | 1 | (6.3) | 0.875 | 0.350 | 9.790 | NA |  |
|  | DRB1 | 15:02 |  | 13 | (9.2) |  | 2 | (6.3) | 0.280 | 0.597 | 16.712 | NA |  | 2 | (12.5) | 0.187 | 0.665 | 18.626 | NA |  | 0 | (0.0) | 1.596 | 0.206 | 5.781 | NA |  |
|  | DRB1 | 16:02 |  | 5 | (3.5) |  | 0 | (0.0) | 1.160 | 0.281 | 7.880 | NA |  | 0 | (0.0) | 0.582 | 0.446 | 12.477 | NA |  | 0 | (0.0) | 0.582 | 0.446 | 12.477 | NA |  |

P_c_, Bonferroni's correction; NA, not applicable; ¥, Fisher exact test

| **Table S5.** Genetic influence of HLA-DQB1 in MERS patients | | | | | | | | | | |  |  |  |  |  |  |  |  |  |  |  |  |  |  |  |  |  |
| --- | --- | --- | --- | --- | --- | --- | --- | --- | --- | --- | --- | --- | --- | --- | --- | --- | --- | --- | --- | --- | --- | --- | --- | --- | --- | --- | --- |
|  |  |  |  | Controls | |  | MERS total | | | | | |  | Moderate/Mild cases | | | | | |  | Severe cases | | | | | |  |
|  | Locus | Alleles |  | n = 142 (%) | |  | n = 32 (%) | | χ^2^ | p-value | P_c_ | OR |  | n = 16 (%) | | χ^2^ | p-value | P_c_ | OR |  | n = 16 (%) | | χ^2^ | p-value | P_c_ | OR |  |
|  | DQB1 | 02:01 |  | 7 | (4.9) |  | 0 | (0.0) | 1.644 | 0.200 | 2.998 | NA |  | 0 | (0.0) | 0.825 | 0.364 | 5.455 | NA |  | 0 | (0.0) | 0.825 | 0.364 | 5.455 | NA |  |
|  | DQB1 | 02:02 |  | 17 | (12.0) |  | 5 | (15.6) | 0.316 | 0.574 | 8.614 | NA |  | 1 | (6.3) | 0.466 | 0.495 | 7.420 | NA |  | 4 | (25.0) | 2.118 | 0.146 | 2.184 | NA |  |
|  | DQB1 | 03:01 |  | 35 | (24.6) |  | 9 | (28.1) | 0.167 | 0.683 | 10.240 | NA |  | 5 | (31.3) | 0.332 | 0.565 | 8.472 | NA |  | 4 | (25.0) | 0.001 | 0.975 | 14.629 | NA |  |
|  | DQB1 | 03:02 |  | 30 | (21.1) |  | 10 | (31.3) | 1.512 | 0.219 | 3.283 | NA |  | 9 | (56.3) | 9.542 | **0.002** | **0.030** | **4.8** |  | 1 | (6.3) | 2.018 | 0.155 | 2.332 | NA |  |
|  | DQB1 | 03:03 |  | 25 | (17.6) |  | 7 | (21.9) | 0.317 | 0.573 | 8.600 | NA |  | 2 | (12.5) | 0.265 | 0.607 | 9.105 | NA |  | 5 | (31.3) | 1.740 | 0.187 | 2.806 | NA |  |
|  | DQB1 | 04:01 |  | 17 | (12.0) |  | 4 | (12.5) | 0.007 | 0.934 | 14.010 | NA |  | 2 | (12.5) | 0.004 | 0.951 | 14.263 | NA |  | 2 | (12.5) | 0.004 | 0.951 | 14.263 | NA |  |
|  | DQB1 | 04:02 |  | 14 | (9.9) |  | 1 | (3.1) | 1.503 | 0.220 | 3.302 | NA |  | 0 | (0.0) | 1.731 | 0.188 | 2.825 | NA |  | 1 | (6.3) | 0.218 | 0.641 | 9.609 | NA |  |
|  | DQB1 | 05:01 |  | 26 | (18.3) |  | 3 | (9.4) | 1.501 | 0.221 | 3.308 | NA |  | 1 | (6.3) | 1.476 | 0.224 | 3.366 | NA |  | 2 | (12.5) | 0.333 | 0.564 | 8.459 | NA |  |
|  | DQB1 | 05:02 |  | 7 | (4.9) |  | 1 | (3.1) | 0.194 | 0.660 | 9.896 | NA |  | 0 | (0.0) | 0.825 | 0.364 | 5.455 | NA |  | 1 | (6.3) | 0.052 | 0.819 | 12.290 | NA |  |
|  | DQB1 | 05:03 |  | 19 | (13.4) |  | 3 | (9.4) | 0.379 | 0.538 | 8.070 | NA |  | 2 | (12.5) | 0.010 | 0.922 | 13.825 | NA |  | 1 | (6.3) | 0.661 | 0.416 | 6.242 | NA |  |
|  | DQB1 | 06:01 |  | 27 | (19.0) |  | 8 | (25.0) | 0.582 | 0.445 | 6.681 | NA |  | 4 | (25.0) | 0.327 | 0.568 | 8.514 | NA |  | 4 | (25.0) | 0.327 | 0.568 | 8.514 | NA |  |
|  | DQB1 | 06:02 |  | 21 | (14.8) |  | 2 | (6.3) | 1.660 | 0.198 | 2.964 | NA |  | 1 | (6.3) | 0.875 | 0.350 | 5.245 | NA |  | 1 | (6.3) | 0.875 | 0.350 | 5.245 | NA |  |
|  | DQB1 | 06:03 |  | 2 | (1.4) |  | 3 | (9.4) | 5.938 | **0.040^¥^** | 5.939 | **7.2** |  | 1 | (6.3) | 1.810 | 0.179 | 2.678 | NA |  | 2 | (12.5) | 7.169 | **0.048^¥^** | 1.346 | **###** |  |
|  | DQB1 | 06:04 |  | 16 | (11.3) |  | 4 | (12.5) | 0.039 | 0.843 | 12.652 | NA |  | 2 | (12.5) | 0.022 | 0.883 | 13.246 | NA |  | 2 | (12.5) | 0.022 | 0.883 | 13.246 | NA |  |
|  | DQB1 | 06:09 |  | 8 | (5.6) |  | 1 | (3.1) | 0.335 | 0.563 | 8.440 | NA |  | 1 | (6.3) | 0.010 | 0.920 | 13.795 | NA |  | 0 | (0.0) | 0.949 | 0.330 | 4.948 | NA |  |

P_c_, Bonferroni's correction; NA, not applicable; ¥, Fisher exact test

| **Table S6.** Genetic influence of HLA-DPB1 in MERS patients | | | | | | | | | | |  |  |  |  |  |  |  |  |  |  |  |  |  |  |  |  |  |
| --- | --- | --- | --- | --- | --- | --- | --- | --- | --- | --- | --- | --- | --- | --- | --- | --- | --- | --- | --- | --- | --- | --- | --- | --- | --- | --- | --- |
|  |  |  |  | Controls | |  | MERS total | | | | | |  | Moderate/Mild cases | | | | | |  | Severe cases | | | | | |  |
|  | Locus | Alleles |  | n = 142 (%) | |  | n = 32 (%) | | χ^2^ | p-value | P_c_ | OR |  | n = 16 (%) | | χ^2^ | p-value | P_c_ | OR |  | n = 16 (%) | | χ^2^ | p-value | P_c_ | OR |  |
|  | DPB1 | 02:01 |  | 64 | (45.1) |  | 16 | (50.0) | 0.255 | 0.613 | 7.359 | NA |  | 9 | (56.3) | 0.723 | 0.395 | 4.742 | NA |  | 7 | (43.8) | 0.010 | 0.920 | 11.038 | NA |  |
|  | DPB1 | 02:02 |  | 8 | (5.6) |  | 3 | (9.4) | 0.617 | 0.432 | 5.185 | NA |  | 0 | (0.0) | 0.949 | 0.330 | 3.958 | NA |  | 3 | (18.8) | 3.819 | 0.051 | 0.608 | NA |  |
|  | DPB1 | 03:01 |  | 7 | (4.9) |  | 1 | (3.1) | 0.194 | 0.660 | 7.916 | NA |  | 1 | (6.3) | 0.052 | 0.819 | 9.832 | NA |  | 0 | (0.0) | 0.825 | 0.364 | 4.364 | NA |  |
|  | DPB1 | 04:01 |  | 22 | (15.5) |  | 6 | (18.8) | 0.205 | 0.651 | 7.807 | NA |  | 4 | (25.0) | 0.945 | 0.331 | 3.971 | NA |  | 3 | (18.8) | 0.115 | 0.735 | 8.821 | NA |  |
|  | DPB1 | 04:02 |  | 21 | (14.8) |  | 1 | (3.1) | 3.217 | 0.073 | 0.875 | NA |  | 0 | (0.0) | 2.729 | 0.099 | 1.183 | NA |  | 1 | (6.3) | 0.875 | 0.350 | 4.196 | NA |  |
|  | DPB1 | 05:01 |  | 88 | (62.0) |  | 14 | (43.8) | 3.575 | 0.059 | 0.704 | NA |  | 8 | (50.0) | 0.864 | 0.353 | 4.230 | NA |  | 8 | (50.0) | 0.864 | 0.353 | 4.230 | NA |  |
|  | DPB1 | 09:01 |  | 9 | (6.3) |  | 3 | (9.4) | 0.375 | 0.540 | 6.483 | NA |  | 3 | (18.8) | 3.157 | 0.076 | 0.907 | NA |  | 0 | (0.0) | 1.075 | 0.300 | 3.597 | NA |  |
|  | DPB1 | 13:01 |  | 19 | (13.4) |  | 3 | (9.4) | 0.379 | 0.538 | 6.456 | NA |  | 2 | (12.5) | 0.010 | 0.922 | 11.060 | NA |  | 3 | (18.8) | 0.346 | 0.556 | 6.677 | NA |  |
|  | DPB1 | 14:01 |  | 5 | (3.5) |  | 0 | (0.0) | 1.160 | 0.281 | 3.377 | NA |  | 0 | (0.0) | 0.582 | 0.446 | 5.347 | NA |  | 0 | (0.0) | 0.582 | 0.446 | 5.347 | NA |  |
|  | DPB1 | 17:01 |  | 9 | (6.3) |  | 0 | (0.0) | 2.139 | 0.144 | 1.723 | NA |  | 0 | (0.0) | 1.075 | 0.300 | 3.597 | NA |  | 0 | (0.0) | 1.075 | 0.300 | 3.597 | NA |  |
|  | DPB1 | 38:01 |  | 1 | (0.7) |  | 0 | (0.0) | 0.227 | 0.634 | 7.608 | NA |  | 0 | (0.0) | 0.113 | 0.736 | 8.836 | NA |  | 0 | (0.0) | 0.113 | 0.736 | 8.836 | NA |  |
|  | DPB1 | 47:01 |  | 1 | (0.7) |  | 0 | (0.0) | 0.227 | 0.634 | 7.608 | NA |  | 0 | (0.0) | 0.113 | 0.736 | 8.836 | NA |  | 0 | (0.0) | 0.113 | 0.736 | 8.836 | NA |  |

P_c_, Bonferroni's correction; NA, not applicable; ¥, Fisher exact test

| **No.** | **Name** | **HLA-A** | **HLA-B** | **HLA-Cw** | **DRB1** | **DQB1** | **DPB1** |
| --- | --- | --- | --- | --- | --- | --- | --- |
| **1** | **MCoV02-1** | ***24:02(A24)** | ***46:01(B46)** | ***01:03(Cw1)** | ***09:01** | ***03:01** | ***05:01** |
|  |  | ***24:02(A24)** | ***40:06(B61)** | ***08:01(Cw8)** | ***12:01** | ***03:03** | ***05:01** |
| **2** | **MCoV03-1** | ***02:06(A2)** | ***15:01(B62)** | ***04:01(Cw4)** | ***04:06** | ***03:02** | ***02:01** |
|  |  | ***11:01(A11)** | ***40:06(B61)** | ***08:01(Cw8)** | ***09:01** | ***03:03** | ***05:01** |
| **3** | **MCoV04-1** | ***24:02(A24)** | ***40:02(B61)** | ***07:06(Cw7)** | ***07:01** | ***02:02** | ***02:01** |
|  |  | ***31:01(A31)** | ***44:03(B44)** | ***03:04(Cw10)** | ***14:07** | ***05:03** | ***13:01** |
| **4** | **MCoV05-1** | ***24:02(A24** | ***35:01(B35)** | ***03:04(Cw10)** | ***08:02** | ***03:02** | ***02:01** |
|  |  | ***33:03(A33)** | ***44:03(B44)** | ***14:03** | ***13:02** | ***06:04** | ***04:01** |
| **5** | **MCoV07-2** | ***30:01(A30)** | ***44:03(B44)** | ***06:02(Cw6)** | ***13:01** | ***06:03** | ***04:01** |
|  |  | ***33:03(A33)** | ***47:01(B47)** | ***14:03** | ***13:02** | ***06:04** | ***04:01** |
| **6** | **MCoV11-1** | ***02:01(A2)** | ***40:01(B60)** | ***14:02** | ***08:03** | ***03:03** | ***02:01** |
|  |  | ***24:02(A24)** | ***51:01(B51)** | ***15:02** | ***12:01** | ***06:01** | ***02:01** |
| **7** | **MCoV12(1)-1** | ***02:01(A2)** | ***44:03(B44)** | ***03:03(Cw9)** | ***07:01** | ***02:02** | ***02:02** |
|  |  | ***33:03(A33)** | ***55:02(B55)** | ***07:06(Cw7)** | ***08:03** | ***06:01** | ***13:01** |
| **8** | **MCoV13-1** | ***11:01(A11)** | ***51:01(B51)** | ***01:02(Cw1)** | ***12:01** | ***03:03** | ***02:02** |
|  |  | ***24:02(A24)** | ***51:01(B51)** | ***14:02** | ***14:54** | ***05:02** | ***05:01** |
| **9** | **MCoV14-1** | ***24:02(A24)** | ***40:02(B61)** | ***03:02(Cw10)** | ***08:02** | ***03:02** | ***02:01** |
|  |  | ***33:03(A33)** | ***58:01(B58)** | ***03:04(Cw10)** | ***13:02** | ***06:09** | ***04:01** |
| **10** | **MCoV21-2** | ***11:01(A11)** | ***27:04(B27)** | ***04:01(Cw4)** | ***04:06** | ***03:01** | ***02:01** |
|  |  | ***11:02(A11)** | ***15:01(B62)** | ***12:02** | ***12:02** | ***03:02** | ***05:01** |
| **11** | **MCoV22-2** | ***02:06(A2)** | ***07:05(B7)** | ***08:01(Cw8)** | ***04:06** | ***03:02** | ***02:01** |
|  |  | ***29:01(A29)** | ***40:06(B61)** | ***15:05** | ***09:01** | ***03:03** | ***05:01** |
| **12** | **MERS-3** | ***01:01(A1)** | ***37:01(B37)** | ***06:02(Cw6)** | ***10:01** | ***05:01** | ***02:01** |
|  |  | ***02:01(A2)** | ***48:01(B48)** | ***08:01(Cw8)** | ***15:01** | ***06:02** | ***04:01** |
| **13** | **NMC-01** | ***02:01(A2)** | ***13:01(B13)** | ***03:04(Cw10)** | ***04:03** | ***03:02** | ***03:01** |
|  |  | ***24:02(A24)** | ***52:01(B52)** | ***12:02** | ***15:02** | ***06:01** | ***09:01** |
| **14** | **NMC-04** | ***02:06(A2)** | ***13:02(B13)** | ***06:02(Cw6)** | ***04:03** | ***03:02** | ***02:01** |
|  |  | ***30:01(A30)** | ***48:01(B48)** | ***08:01(Cw8)** | ***04:07** | ***03:02** | ***02:01** |
| **15** | **NMC-07** | ***02:07(A2)** | ***46:01(B46)** | ***01:02(Cw1)** | ***08:03** | ***06:01** | ***04:01** |
|  |  | ***30:01(A30)** | ***47:01(B47)** | ***06:02(Cw6)** | ***13:01** | ***06:03** | ***05:01** |
| **16** | **NMC-08** | ***01:01(A1)** | ***37:01(B37)** | ***06:02(Cw6)** | ***10:01** | ***05:01** | ***02:01** |
|  |  | ***02:01(A2)** | ***48:01(B48)** | ***08:01(Cw8)** | ***15:01** | ***06:02** | ***04:01** |
| **17** | **NMC-10** | ***02:01(A2)** | ***46:01(B46)** | ***01:02(Cw1)** | ***08:03** | ***03:01** | ***02:01** |
|  |  | ***02:07(A2)** | ***51:01(B51)** | ***14:02** | ***14:03** | ***06:01** | ***09:01** |
| **18** | **NMC-12** | ***02:01(A2)** | ***15:11(B75)** | ***03:03(Cw9)** | ***04:05** | ***04:01** | ***05:01** |
|  |  | ***33:03(A33)** | ***44:03(B44)** | ***14:03** | ***13:02** | ***06:04** | ***05:01** |
| **19** | **NMC-13** | ***02:06(A2)** | ***40:02(B61)** | ***03:04(Cw10)** | ***04:06** | ***03:01** | ***05:01** |
|  |  | ***02:06(A2)** | ***51:01(B51)** | ***14:02** | ***14:03** | ***03:02** | ***05:01** |
| **20** | **NMC-14** | ***33:03(A33)** | ***15:01(B62)** | ***01:02(Cw1)** | ***07:01** | ***02:02** | ***04:01** |
|  |  | ***33:03(A33)** | ***44:03(B44)** | ***07:06(Cw7)** | ***13:02** | ***06:04** | ***13:01** |
| **21** | **NMC-02** | ***02:07(A2)** | ***35:01(B35)** | ***01:02(Cw1)** | ***08:03** | ***03:03** | ***02:02** |
|  |  | ***24:02(A24)** | ***46:01(B46)** | ***03:03(Cw9)** | ***09:01** | ***03:03** | ***02:02** |
| **22** | **NMC-03** | ***24:02(A24)** | ***44:02(B44)** | ***01:02(Cw1)** | ***04:05** | ***04:01** | ***02:01** |
|  |  | ***31:01(A31)** | ***54:01(B54)** | ***05:01(Cw5)** | ***13:01** | ***06:03** | ***05:01** |
| **23** | **NMC-06** | ***24:02(A24)** | ***07:02(B7)** | ***07:02(Cw7)** | ***04:05** | ***04:01** | ***05:01** |
|  |  | ***24:02(A24)** | ***52:01(B52)** | ***12:02** | ***15:02** | ***06:01** | ***09:01** |
| **24** | **NMC-15** | ***24:02(A24)** | ***38:02(B38)** | ***07:02(Cw7)** | ***04:03** | ***03:01** | ***05:01** |
|  |  | ***31:01(A31)** | ***39:01(B39)** | ***07:02(Cw7)** | ***08:03** | ***03:02** | ***13:01** |
| **25** | **NMC-16** | ***24:02(A24)** | ***40:01(B60)** | ***04:01(Cw4)** | ***04:05** | ***02:02** | ***05:01** |
|  |  | ***33:03(A33)** | ***44:03(B44)** | ***07:06(Cw7)** | ***07:01** | ***04:01** | ***13:01** |
| **26** | **NMC-17** | ***24:02(A24)** | ***07:02(B7)** | ***01:02(Cw1)** | ***01:01** | ***03:01** | ***04:02** |
|  |  | ***24:02(A24)** | ***59:01(B59)** | ***07:02(Cw7)** | ***12:02** | ***05:01** | ***05:01** |
| **27** | **NMC-18** | ***11:01(A11)** | ***15:07(B62)** | ***03:03(Cw9)** | ***08:03** | ***03:01** | ***05:01** |
|  |  | ***31:01(A31)** | ***55:04(B55)** | ***03:03(Cw9)** | ***12:02** | ***06:01** | ***41:01** |
| **28** | **Seoul-01** | ***03:01(A3)** | ***13:02(B13)** | ***03:413** | ***07:01** | ***02:02** | ***02:01** |
|  |  | ***30:01(A30)** | ***15:11(B75)** | ***06:02(Cw6)** | ***08:02** | ***04:02** | ***02:01** |
| **29** | **Seoul-02** | ***02:01(A2)** | ***15:01(B62)** | ***03:03(Cw9)** | ***04:06** | ***03:01** | ***02:01** |
|  |  | ***26:01(A26)** | ***35:01(B35)** | ***08:01(Cw8)** | ***12:10** | ***03:02** | ***02:01** |
| **30** | **Seoul-03** | ***26:01(A26)** | ***15:11(B75)** | ***03:03(Cw9)** | ***08:03** | ***05:03** | ***02:01** |
|  |  | ***33:03(A33)** | ***40:03(B61)** | ***03:04(Cw10)** | ***14:05** | ***06:01** | ***02:01** |
| **31** | **Seoul-04** | ***02:06(A2)** | ***51:01(B51)** | ***14:02** | ***09:01** | ***03:01** | ***02:01** |
|  |  | ***02:06(A2)** | ***51:01(B51)** | ***14:02** | ***12:02** | ***03:03** | ***05:01** |
| **32** | **Seoul-06** | ***11:01(A11)** | ***40:02(B61)** | ***03:03(Cw9)** | ***14:05** | ***05:03** | ***05:01** |
|  |  | ***24:02(A24)** | ***54:01(B54)** | ***03:03(Cw9)** | ***14:54** | ***05:03** | ***05:01** |
